# Removal of Methylene Blue from Wastewater by Waste Roots from the Arsenic-Hyperaccumulator *Pteris vittata*: Fixed Bed Adsorption Kinetics

**DOI:** 10.3390/ma16041450

**Published:** 2023-02-09

**Authors:** Leone Mazzeo, Davide Marzi, Irene Bavasso, Vincenzo Piemonte, Luca Di Palma

**Affiliations:** 1 Department of Chemical Engineering Materials & Environment, Sapienza University of Rome, Via Eudossiana, 18, 00184 Rome, Italy; 2 Department of Engineering, University Campus Biomedico of Rome, Via Alvaro del Portillo, 21, 00128 Rome, Italy; 3 Department of Biology and Biotechnology “Charles Darwin”, Sapienza University of Rome, Piazzale Aldo Moro 5, 00185 Rome, Italy

**Keywords:** *pteris vittata*, fixed-bed, adsorption, modelling, methylene blue, water treatment

## Abstract

Phytoremediation of arsenic-contaminated water was successfully conducted by means of the perennial fern *Pteris vittate*, which is an arsenic-hyperaccumulator plant able to grow in hydroponic cultures. In order to avoid the costs linked to the disposal of As-contaminated biomass, in this work, *Pteris vittata* waste roots were tested as a low-cost bio-adsorbent for the removal of methylene blue (MB) from water in a fixed-bed adsorption configuration. As a matter of fact, methylene blue can negatively impact the growth and health of algae and plants by blocking light from reaching them in water, which can alter their normal biological processes. Previous works have already shown the potentiality of such material toward the uptake of methylene blue; however, all the studies conducted were just focused on batch-mode experiments. In this work, column runs were carried out at 20 °C, evaluating the bed void fraction for each test and hence estimating the apparent density of the material (300 g/L). The breakthrough curves collected were fitted by means of a mathematical model based on the linear driving force (LDF) approximation to obtain information on the mass transfer mechanism occurring in the system. A relation for the product between the LDF mass transfer coefficient and the solid specific surface ($k_{LDF}a_{s}$) with respect to the Reynolds (Re) dimensionless number was obtained ($k_{LDF}a_{s}=0.45Re$). The range of validity of such expression was Re<0.025. Its applicability was deeply discussed: in such conditions, the technology is ready to be tested at larger scales.

## 1. Introduction

Water is a fundamental element for life on Earth and it is crucial for social and economic development. The principal users of water are the agriculture, domestic and industrial sectors. These human activities produce polluted water effluents, the uncontrolled release of which may induce severe environmental damage and the contamination of freshwater reservoirs. Moreover, it was estimated that climate change coupled with the exponential growth of the population will lead to an overall decrease in freshwater availability [1]. Waste-water treatment plays, then, a fundamental role in maintaining water bodies in good ecological status and achieving the sustainable management of water as set by the United Nations’ 2030 Agenda for Sustainable Development [2].

Dyes are a class of contaminants of major concern since they are easily detected even at concentrations < 1 ppm and they can lead to allergies, dermatitis and even cancers through contact with the gastrointestinal tract, skin and lungs [3]. Moreover, dyes can perturb the biological activities of algae by hindering light penetration in water. Approximately 7 × 10^5^ ÷ 1 × 10^6^ tons [4,5,6] of dyes are produced each year and studies have shown that about 10–15% of this amount is lost during the dyeing process [7,8]. The textile, pulp and paper and tannery industries are the main users of dyes and they generate 120, 150 and 28 m^3^ of wastewater per ton of fibres, paper and rawhide, respectively [6].

Primary and secondary wastewater (e.g., activated sludge in a sequential batch reactor [9]) treatment alone is often not enough for the removal of diluted compounds. For this reason, tertiary treatment is required. Filtration techniques such as reverse osmosis (RO) or advanced oxidation processes (AOPs) (e.g., degradation of methylene blue under visible [10] and UV light [11]) are, then, employed for this scope. However, the former technique produces an undesired concentrated sludge/brine that should be further processed in order to avoid environmental concerns due to its disposal [12,13]; while the latter require severe control of the working conditions (e.g., pH) to achieve a complete mineralization/degradation of the target compounds [5].

Adsorption has a wide application for the removal of very diluted pollutants and appears to be a valid alternative to the majority of dye-rich wastewater treatment technologies due to its ease of operation and its intrinsic characteristic of not generating toxic effluents. However, the most commonly adopted solid adsorbents such as active carbons or zeolites have to sustain high activation and synthesis costs, respectively. A valid attempt to abate such costs was to use waste streams as raw material for adsorbent synthesis (e.g., active carbons from sewage sludge [14]). Another recent approach, following circular economy principles, is to perform adsorption with new low-cost and environmentally friendly bio-adsorbents obtained from agro-industrial waste.

*Pteris vittata* (PV) is a herbaceous fern with tropical and subtropical distribution. Several studies have documented the ability of PV to remove large amounts of arsenic (As) (up to 23.000 mg/kg) from soil [15] and directly from the water in hydroponic culture systems [16,17]. Multiple phytoremediation cycles were also successfully conducted [18] showing that PV does not release any of the previously accumulated As.

The most important aspect that limits the development of phytoremediation is the disposal of contaminated waste biomass, which constitutes a major cost [19]. Although As-rich PV was already subjected to gasification [20], hydrothermal liquefaction [21] and combustion [22], in order to produce income from the generation of valuable products (e.g., bio-oil, biochar, biogas, heat and As-rich ash), the process still resulted as economically inconvenient. For this reason, several alternatives were investigated, such as the extraction of bio-active compounds (BACs) (i.e., flavonoids, proanthocyanidins, phenolic acids) [23] or PV utilization as reinforcement for polymer composites [24].

An alternative approach was instead proposed in the work of Mazzeo et al. [25], where the waste roots of *Pteris vittata* were used as a novel bio-adsorbent for wastewater treatment and in particular for the uptake of methylene blue (MB). The latter was selected as a representative cationic dye due to its frequent use in the textile industry [26] and in scientific papers, making the comparison of its effectiveness with respect to other bio-adsorbents made from agricultural waste easier [27,28]. Furthermore, in addition to environmental concerns [29], MB, when inhaled, can cause difficulty breathing and, when it comes into contact with the eyes, it can cause burning. When ingested, it can cause symptoms such as nausea, vomiting, excessive sweating, mental confusion and a condition called methemoglobinemia [30]. In the abovementioned paper (Mazzeo et al. [25]), the adsorption tests were performed only in batch mode. Hence, this work aims to make a step forward in the utilization of PV roots as a novel bio-adsorbent, performing fixed-bed column tests for the removal of MB from water. The thermodynamic results of the work of Mazzeo et al. [25] were used as input to the mathematical model used to fit the experimental breakthrough curves. In this work, crucial information for the comprehension of the mass transfer behaviour of the system were obtained, making it possible to assess the feasibility of a full-scale process unit.

## 2. Materials and Methods

### 2.1. Chemicals

Methylene blue (C_16_H_18_ClN_3_S; dye content ≥ 82.0%) was purchased from Sigma Aldrich (St. Louis, MI, USA) and used without any further purification.

### 2.2. Bio-Adsorbent Preparation

Spores of the Chinese fern brake *Pteris vittate* (PV) were obtained from ferns collected at the Botanical Garden of La Tuscia University (Viterbo, Italy) and moved to a greenhouse for propagation. The spores were resuspended in water, sown in pots filled with soil and clay and covered with a plastic wrap; the pots were sprayed with distilled water to maintain humidity. After two months, the young ferns of about 2 cm in height were moved to individual rockwool cubes and placed in mini greenhouses for 4–6 weeks. Thus, the ferns were moved to net pots filled with expanded clay and placed in 15L tanks of tap water for hydroponic culture. Each tank hosted 12 ferns, and after 4–5 months, the roots occupied most of the space in the tank; thus, the roots were cut and harvested [18]. The waste roots were pretreated before the adsorption tests, following the procedure reported in the work of Mazzeo et al. [25]. Briefly, the PV roots were washed by mixing 2 g of them in 250 mL of demineralized water at room temperature in a 500 mL beaker. This mixture was vigorously agitated with a magnetic stirrer for 1 h. After washing, the roots were dried at 50 °C for 24 h. Once pretreated, the roots were cut in order to reduce their length to a desired value. The latter was not the same for all the tests: the corresponding root length of each test was reported in the next section.

### 2.3. Experimental Set-Up and Column Tests

**Figure 1:**
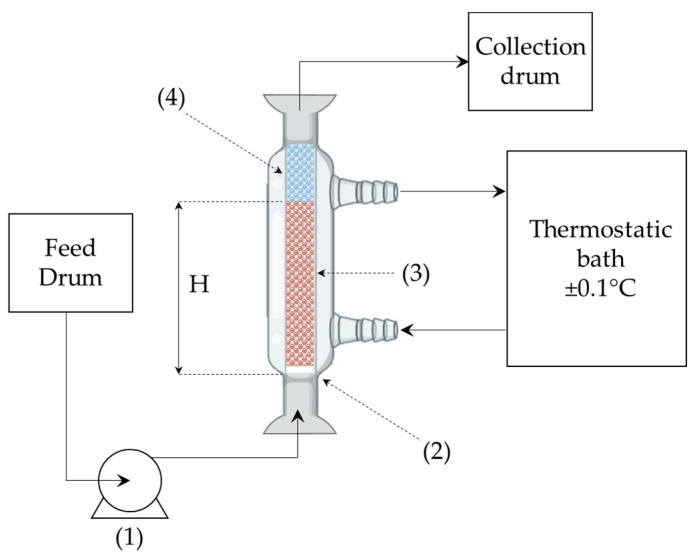
Experimental apparatus: (1) peristaltic pump; (2) glass porous filter; (3) solid adsorbent; (4) glass spheres.

Fixed-bed adsorption runs were carried out in a glass column (1 cm internal diameter). At the bottom of the column was located a porous glass septum used to support the solid adsorbent. The column was filled with 0.232 g of solid in all tests, but in T1 and T2 the length of the roots was around 0.8 cm, while in the others it was 0.4 cm, hence the bed height (H) varied accordingly as reported in Table 1. A liquid inlet solution of methylene blue (MB) was continuously fed to the bottom of the column by means of a variable-speed peristaltic pump. This configuration was selected to assure the complete wetting of the adsorbent bed since it generates the establishment of a liquid hold-up or, in other words, it fully fills the column of liquid during the adsorption tests. As a matter of fact, it was observed that (in the range of the fluid flow rate analysed 5.9–13.6 mL/min), if the liquid had been fed to the top of the column, droplets of liquid would have been observed wetting the bed without guaranteeing its complete coverage. It is worth noting that upflow promotes the drag of solid particles, especially when the intrinsic density of the solid is lower than the liquid one (carbonaceous materials happen to be so with respect to water). In order to prevent the movement of particles within the bed, it is suggested [31] to set the fluid upflow rate to less than about 80% of the minimum fluidization velocity. However, in this study, the top of the adsorbent bed was covered by two layers of glass spheres of 1 mm and 50 mm in diameter, respectively, which avoided solid drag. For this reason, higher velocities than the minimum fluidization one were reached. The column was equipped with a thermostat jacket in order to keep the temperature at 20 ± 0.1 °C. Before the beginning of each test, the bed was rinsed with distilled water for 1 h to remove dust or other residue and to achieve complete wetting of the solid surface. A summary of the operating conditions used in the experimental runs is presented in Table 1, while a detailed scheme of the experimental apparatus is given in Figure 1. In all the tests, the concentration of MB in the liquid solution was calculated using a PG Instruments (United States) T80+ UV/Vis spectrophotometer (with glass cells of 1 cm path length) at a wavelength of 664 nm. The experimental data were fitted using a mathematical model (discussed in the following section) employing the process simulator gProms (Process System Enterprises, London, UK) to estimate the parameters of the model and detect the controlling resistance of the system.

### 2.4. Bed Void Fraction and Solid Apparent Density Estimation

The estimation of the bed void fraction is crucial to avoid mistakes in the comprehension of the mass transfer conditions occurring in the adsorption process. The method developed in this study is based on measuring the permanence time of the liquid in the column. The interstitial velocity is higher than the superficial one; thus, the observed residence time in the unit is lower than the one that would exist without packing. According to such consideration, when the packed bed was filled for the first time with distillate water for the rinse, the effective residence time of the liquid was measured ($\tau^{*}$). Setting the liquid flow rate (Q) and knowing the column height (H) and diameter ($R_{c}$), the bed void fraction (ε) was calculated as follows

(1) $$\varepsilon =\frac{Q\tau^{*}}{H\pi R_{c}^{2}}$$

Once the value of the bed void fraction was obtained, it was also possible to evaluate the material’s apparent density ($\rho_{s}$) using Equation (2), where $M_{s}$ is the amount of solid loaded in the column. This step is of fundamental importance when dealing with waste materials since their properties are often unknown

(2) $$\rho_{s}=\frac{M_{s}}{\left(1-\varepsilon \right)H\pi R_{c}^{2}}$$

## 3. Results and Discussion

### 3.1. Bio-Adsorbent Preparation

The method described in Section 2.4 was applied to all the tests and the results were collected in Table 2. The validity of the method was verified since the resulting density was almost the same for every test. The average density was calculated to be 299 g/L and this value will be used in the numerical simulation of the continuous kinetic modelling. The test T5 was used just as an additional run for the estimation of the solid density, while for all the other tests, the kinetic behaviour was also investigated.

### 3.2. Column Tests

The model used to describe the evolution of the MB concentration during the time along the column length was based on the following assumptions:

- the process is isothermal;
- both convection and axial dispersion were considered in the liquid phase: the well-known “longitudinal dispersed plug flow model” or simply the “dispersion model” [32] was adopted to take into account the fluid-dynamic deviations from an ideal plug-flow motion;
- the mass transfer of MB from the liquid to the solid phase was described by means of the well-established [33] linear driving force (LDF) approximation.

The unsteady state, one-dimensional material balance for a cylindrical section that describes the transient adsorption of MB in the fixed-bed column, was then reported in Equation (3) coupled with a material balance in the solid phase described by Equation (4).

(3) $$\varepsilon \frac{\partial C}{\partial t}+\langle v\rangle \frac{\partial C}{\partial z}=E_{z}\frac{\partial^{2}C}{\partial z^{2}}-\left(1-\varepsilon \right)\rho_{s}\frac{\partial q}{\partial t}$$

(4) $$\frac{\partial q}{\partial t}=k_{LDF}a_{s}\left(q^{*}-q\right)$$

Here, ε is the bed void fraction, 〈v〉 is the superficial velocity, $a_{s}$ is the specific surface area of the solid, $k_{LDF}$ is the LDF mass transfer coefficient, C is the concentration of MB in the liquid phase, q is the solid surface concentration of MB, $E_{z}$ is the effective longitudinal dispersion coefficient and $q^{*}$ is the hypothetical solid surface concentration of MB in equilibrium with the liquid bulk. The boundary conditions of such a set of equations are

(5) $${\frac{\partial C}{\partial z}|}_{z=H}=0;\;\;\;{C|}_{z=0}=C_{in}$$

where $C_{in}$ is the concentration of the inlet MB solution, while the initial conditions are

(6) C(z)=0;  0<z<Lq(z)=0; 0≤z≤L

The effective longitudinal dispersion coefficient was predicted by manipulating the correlation of Chung and Wen [32] for fixed-bed columns. The latter provides a relation between the dimensionless Peclét (Pé) and Reynolds (Re) numbers, as shown in Equation (7), together with its range of validity. The effective longitudinal dispersion coefficient $E_{z}$ was then calculated from the definition of Pé (Equation (8)). For completeness, the definition of Re is also given in Equation (8).

(7) $$Pé=\frac{H}{d_{p}}\left[0.2+0.011Re^{0.48}\right]\;\;;\;10^{-3}<R<10^{3}$$

(8) $$Pé=\frac{H\langle v\rangle }{E_{z}};Re=\frac{\rho_{L}\langle v\rangle d_{p}}{\mu_{L}}$$

In Equation (8), $\rho_{L}$ represents the liquid density, $\mu_{L}$ is the liquid viscosity and in Equations (7) and (8) $d_{p}$ is the diameter for spherical solid particles. In this case, the shape of the solid was assumed to be cylindrical; hence, $d_{p}$ was substituted by an equivalent diameter ($d_{e}$) calculated as shown in Equation (9).

(9) $$d_{e}=2\sqrt[{3}]{\frac{3}{4}R_{r}^{2}L_{r}}$$

where $L_{r}$ and $R_{r}$ are the length and the radius of the roots respectively. The equivalent diameter was calculated to be 0.078 cm and 0.062 cm for T1, T2 and T3, T4, respectively.

The product between the LDF mass transfer coefficient and the specific surface area of the solid ($k_{LDF}a_{s}$) was used as an adjustable parameter to perform data fitting using the maximum likelihood method as an estimation technique. The error variance was considered constant since no dilutions were required in the range of concentrations adopted during the kinetic runs.

The thermodynamic equilibrium was described using the Langmuir isotherm according to the results obtained in the work of Mazzeo et al. [25]. Thus, the value of the maximum adsorbed capacity for the unit mass of adsorbent ($q_{max}$) and the Langmuir constant b were set as 112.34 mg/g and 0.62 L/mg, respectively. A summary of the common parameters to all the kinetic tests and their corresponding values used in the theoretical model are listed in Table 3.

**Figure 2:**
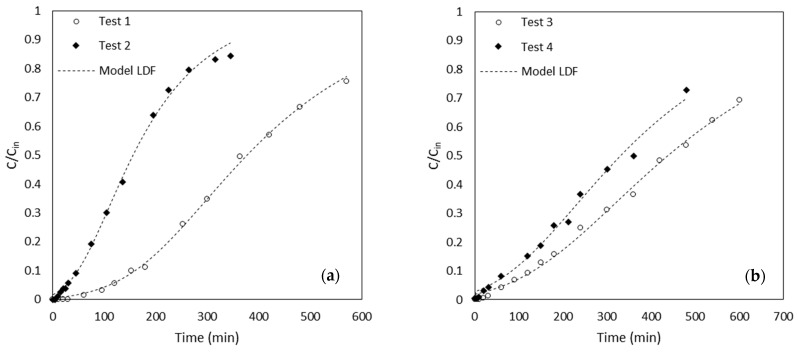
MB experimental and predicted breakthrough curves at 20 °C for *Pteris Vittata* roots of 0.8 cm (**a**) and 0.4 cm (**b**) in length.

The experimental results together with the model-predicted MB breakthrough curves are reported in Figure 2a,b for the roots of lengths of 0.8 cm and 0.4 cm, respectively. It was observed that the model well represents the behaviour of the system; moreover, the goodness of fit for each estimation was evaluated with the chi-squared test ($\chi^{2}$) test, selecting a significance level of α = 0.05 (data not shown).

**Figure 3:**
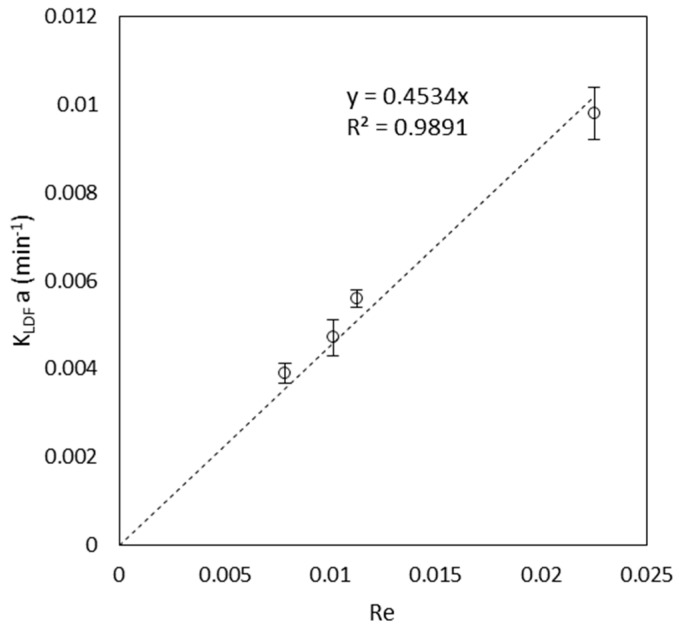
Plot of the estimated parameter $k_{LDF}a_{s}$ versus the Reynolds number Re. The error bars correspond to the confidence interval with a significance level of α=0.02.

The values of $k_{LDF}a_{s}$ estimated are reported in Figure 3 with their corresponding confidence interval versus the Reynolds number. Values of the same order of magnitude $(\sim 10^{-3}\frac{1}{min}$) of $k_{LDF}a_{s}$ were reported by de Araujo et al. [34], which studied the adsorption of different dyes (including MB) on an agar-graphene oxide bio-composite hydrogel.

From Figure 3, it is immediately noticeable that the estimated parameters seem to lie on a straight line passing from the axis of origin, and this is more than just intuition. As a matter of fact, many correlations in the estimation of the mass transfer coefficient proposed in the literature [35,36,37] predict that no mass transfer should occur when Re→0, which is obviously not true. This topic was deeply discussed by Ruthven in his book “Principle of adsorption and Adsorption processes” [31]. It concluded that an erroneous underestimation of the axial dispersion coefficient leads to erroneous low values for the mass transfer coefficient. Then, it is possible that, in this work, the expression of Chung and Wen provides an underestimation of the real effect of longitudinal dispersion. Unfortunately, also the applications of other expressions such as the one of Leitão et al. [38] led to similar results (data not shown). According to such consideration, a linear regression starting from the axis origin was used to correlate the estimated data obtaining the relation reported in Equation (10), which showed an $R^{2}=0.989$.

(10) $$k_{LDF}a_{s}=0.45Re$$

This result clearly evidences a strong dependence of the LDF mass transfer coefficient from the liquid flow rate, thus proving that the external mass transfer resistance plays a key role in the kinetics of the adsorption process. Moreover, $k_{LDF}a_{s}$ seems also not to be affected by the bed void fraction variation nor by the length of the roots.

**Figure 4:**
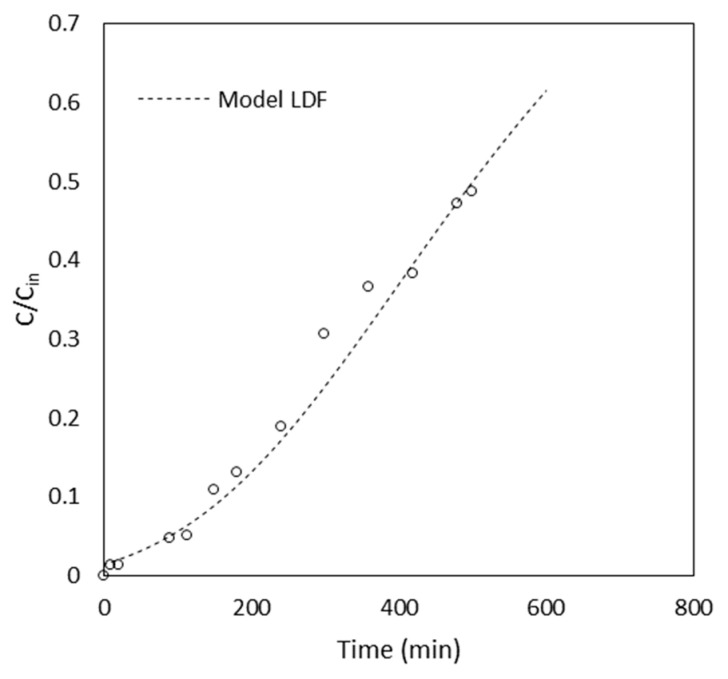
MB experimental and predicted breakthrough curve at 20 °C for *Pteris Vittata* roots of 0.8 cm in length (test V1).

At this point, in order to verify and validate the expression of Equation (10), an additional column run (V1) was performed at T=20 ± 0.1 °C with the aim of predicting the experimental trend. The particular operating conditions of test V1 are reported in Table 4 (in the model, the values shown in Table 3 were also used). The result is provided in Figure 4, showing a good prediction of the experimental points.

**Figure 5:**
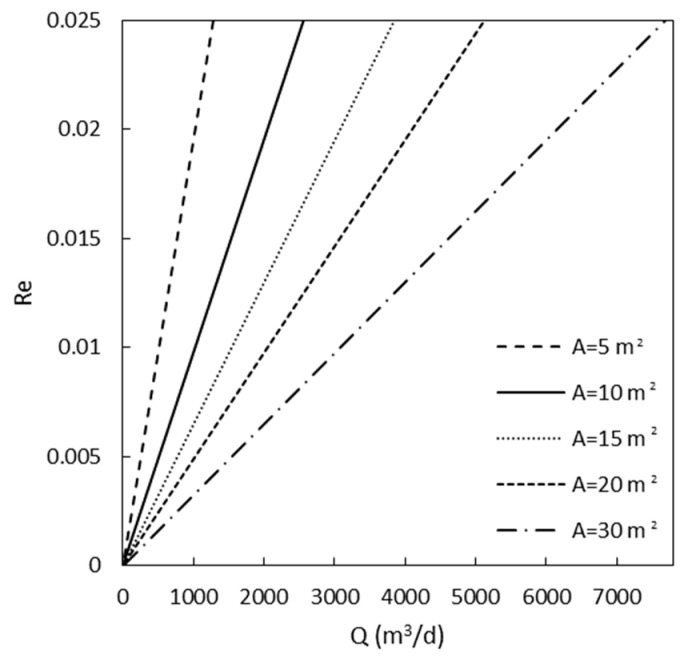
Reynolds number vs. liquid flow rate and column cross-sectional area.

It is possible to affirm then that, in the range of Re explored (Re<0.025), the expression of Equation (10) is reliable for the prediction of the system behaviour. It is interesting now to understand also the applicability of this Equation for real wastewater treatment scenarios. According to the book by Cecen and Aktas [39], the range of liquid flow rates ($Q_{L}$) in industrial wastewater plants lies between 19–16,000 m^3^/day, while typical values of cross-sectional areas (A) for adsorption columns are in the range of 5–30 m^2^. All the possible combinations of ($Q_{L}$) and A that resulted in Re<0.025 (assuming as a reasonable value the length of the roots equal to 1 cm) were calculated with Equation (8) and plotted in Figure 5. The latter constitutes an orientation map for real applications of *Pteris Vittata* as a solid adsorbent for MB removal using the expression $k_{LDF}a=0.45Re$.

## 4. Conclusions

In this work, *Pteris vittata* was tested as a bio-adsorbent in a column operation mode for the removal of MB from wastewater. First of all, the bed void fraction for every test was estimated and, later, the apparent density of the material was calculated to be 299.2 g/L. Then, column breakthrough curves were obtained experimentally and fitted using a mathematical model that took into account the effects of axial dispersion coupled with convection and assumed the linear driving force (LDF) approximation as valid to describe the mass transfer from the liquid to the solid phase. According to the results obtained from fitting, an important contribution of the external liquid film resistance on the mass transfer was observed and an expression for its evaluation ($k_{LDF}a_{s}=0.45Re$) was derived. This was validated by an additional run, the experimental data of which were precisely predicted by the model. However, the equation is only valid for Re < 0.025. Flow rates and cross-sectional areas where the expression can be applied were also provided.

The results of this work could be used to perform larger scale tests, but further studies are needed to extend the reliability of the expression for the mass transfer coefficient to higher values of Re.

## Figures and Tables

**Table 1:** Operating conditions of experimental runs. (T=20 ± 0.1 °C, $M_{s}=0.232\;g$ ).

Test	H (cm)	Q (ml/min)	$C_{in}\;\left(mg/L\right)$
T1	4.5	6.8	6.5
T2	4.5	13.6	7.8
T3	3	5.9	6.1
T4	3	7.7	5.9

**Table 2:** Evaluation of the solid intrinsic density of *Pteris vittata* based on the bed void fraction estimation for each test. ($M_{s}=0.232\;g$ ).

Test	H (cm)	Q (ml/min)	$\tau^{*}\;\left(s\right)$	ε	$\rho_{s}\;\left(g/L\right)$
T1	4.5	6.90	24	0.781	300.3
T2	4.5	7.50	22	0.778	296.5
T3	3	7.07	13.3	0.665	294.4
T4	3	7.95	12	0.675	303.2
T5	3	7.05	13.5	0.674	301.7
**Average** ± **DS**					299.2 ± 3.6

**Table 3:** Values of the common parameters of all the kinetic tests used in theoretical model.

Parameter	Description	Value (UOM)	Reference
$\rho_{s}$	Solid apparent density	299.2 g/L	[This work]
$q_{max}$	Maximum adsorption capacity	112.34 mg/g	[25]
b	Langmuir constant	0.62 L/mg	[25]

**Table 4:** Operating conditions of test V1.

Parameter	Description	Value (UOM)	Notes
$M_{s}$	Mass of solid	0.228 g	-
Q	Flow rate	6.3 mL/min	-
$C_{in}$	Inlet MB concentration	4.1 mg/L	-
H	Bed height	2.7 cm	-
$d_{e}$	Equivalent particle diameter	0.078 cm	-
ε	Bed void fraction	0.664	Equation (1)
Re	Reynolds number	0.0104	Equation (8)
$k_{LDF}a$	Product of LDF mass transfer coefficient with the specific surface area of PV roots	4.68 × 10^−3^ 1/min	Equation (10)

## Data Availability

Not applicable.
